# The Development and Field Evaluation of an IoT System of Low-Power Vibration for Bridge Health Monitoring

**DOI:** 10.3390/s19051222

**Published:** 2019-03-11

**Authors:** Xinlong Tong, Hailu Yang, Linbing Wang, Yinghao Miao

**Affiliations:** 1 National Center for Materials Service Safety, University of Science and Technology Beijing, Beijing 100083, China; maniga@163.com (X.T.); yanghailu@ustb.edu.cn (H.Y.); 2 Joint USTB-Virginia Tech Lab on Multifunctional Materials, USTB, Beijing 100083, China; 3 Virginia Tech, Blacksburg, VA 24061, USA

**Keywords:** bridges, Internet of Things, sensors, gateway

## Abstract

Bridge safety is important for the safety of vehicles and pedestrians. This paper presents a study on the development of a low-power wireless acceleration sensor and deployment of the sensors on a wireless gateway and cloud platform following the Internet of Things (IoT) protocols for bridge monitoring. The entire system was validated in a field test on the Chijing bridge in Shanghai. Field evaluations indicated that the developed IoT bridge monitoring system could achieve the functions of real-time data acquisition, transmission, storage and analytical processing to synthesize safety information of the bridge. The demonstrated system was promising as a complete, practical, readily available, low-cost IoT system for bridge health monitoring.

## 1. Introduction

Bridges support traffic loading and play an important role in the transportation system. The safety of bridges is tightly related to the safety of vehicles and pedestrians. In recent years, bridge collapses have occurred frequently, which have resulted in not only huge economic losses and negative social effects but have also caused casualties. In practice, the service life of some bridges is less than their design life [1,2]. There are different degrees of damage and operational errors in many bridges that are still in service, creating various potential risks that threaten the safety of pedestrians and vehicles all the time. Therefore, the monitoring and evaluation of bridge performance in real-time is important to understand the conditions of bridge health and improve the safety of bridges [3,4].

At present, bridge health monitoring is usually carried out by laying wired sensors, and data must be collected on-site regularly [5,6], which does not meet the requirements of long-term, real-time, and on-line automated monitoring for structure parameters. Manual monitoring has obvious shortcomings: (1) Monitoring is typically not regular and some results are obtained by empirical estimation; (2) due to the limitation of data collection frequency, testing results often lag behind the damage of the bridge [7], and there are no early warnings of damage; (3) due to the lack of real-time monitored data, it is difficult to understand the development of damage. With the advance of the Internet of Things (the IoT), health monitoring of infrastructure has an opportunity for technological advances [8,9]. By making use of IoT, better interconnection among all infrastructure objects can be established to enhance data collection, transmission and processing. In fact, data collection, transmission and processing are the three frame components of an IoT system [10].

Lazarescu and Mihai T address all phases of the practical development from scratch of a full customized WSN platform for environmental monitoring [11]. Aono, Kenji extended the coverage of IoT to monitor the health of different segments of a large civil infrastructure like highway pavements, buildings or a multi-span bridge by incorporating sensing capabilities in passive radio frequency identification (RFID) tagging technology [12]. Shuai Guo has analyzed the damage information of bridges via a neural network to identify damage locations and assess damage degrees using the difference of deflection between the damaged and the intact structures as the input to the neural network [13].

The development and application of an IoT bridge health monitoring system includes the following elements: (1) Design and integration of wireless low-power acceleration sensors, wireless data gateway, and cloud platform terminal following the IoT protocols; (2) development of field installation and the layout plan for sensors and gateways according to the bridge structure [14]; so that the sensors can collect desired data automatically and transmit data to the corresponding gateway; and (3) design of a data transmission system to transmit data to the cloud platform [15,16,17]. Bridge managers assess data during the bridge operation period from the cloud platform in real time, and understand the health conditions of the bridge [18,19]. 

Through the literature review, most of the bridge monitoring was conducted using a wiring system. Some of the IoT based system was also tried in practice, but it is not yet standardized. And the energy supply for long-term monitoring is still limited for the wireless system. This paper presents the development of a wireless acceleration sensor with low power consumption and low cost, the integration of a wireless gateway with low power consumption and self-power supply, and a cloud platform with data upload and download, data storage and visualization following the IoT protocols. Power consumption can be significantly reduced by introducing the sleep–wake up mechanism into the system. Additionally, the performance of the wireless acceleration sensor including a wireless cantilever beam sensor and wireless gateway was tested evaluated. The data acquisition and wireless transmission capacity was tested on an in-service bridge. 

Tests have indicated that the IoT-based bridge health monitoring system designed in this study achieved real-time and regular data collection, transmission, storage, and health state analysis. Multi-bridge unified monitoring could be achieved by adding sensors and gateways in the monitoring system. The feasibility of data acquisition and wireless transmission with a piezoelectric cantilever beam was verified by testing. The piezoelectric cantilever beam could harvest the vibration energy and convert it into electrical energy, which presents a capability for developing a wireless self-powered sensor. The IoT system designed in this paper was applied to the new bridge; at the time of the study there was no monitoring and analysis of damage to the bridge.

## 2. Design of Wireless Sensor for Monitoring Nodes

The sensor of the front-end monitoring node should be different from the traditional wired monitoring system in order to form an IoT based bridge health monitoring system. It should be convenient for data transmission, sensor layout and management, and therefore wireless sensors should be selected. Each wireless sensor corresponds to a monitoring node when the IoT system is operating. The data collected by the sensor will be transmitted to a gateway through the built-in wireless module, and then uploaded in real-time to the cloud platform by the gateway [20]. 

### 2.1. Low Power Wireless Acceleration Sensor

#### 2.1.1. Design of Wireless Acceleration Sensor

**Figure 1:**
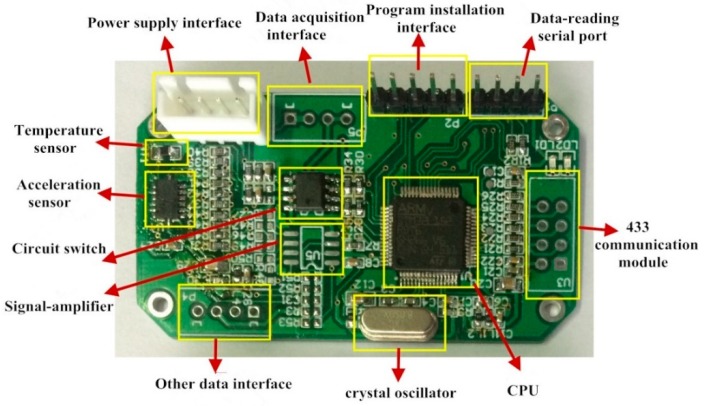
The wireless acceleration sensor structure.

**Figure 2:**
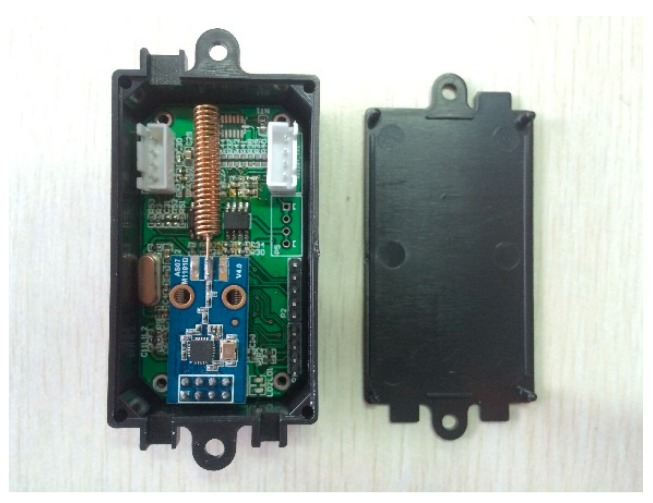
The packaged wireless acceleration sensor.

This study focuses on a low-power, high-precision, wireless transmission, high communication accuracy acceleration sensor which can be suitable for bridge structural monitoring. The acceleration sensor is heavily based on computer technology and communication technology to improve system stability, reliability, and low-power performance. The system consists of a 32-bit low-power ARM (Advanced RISC (Reduced Instruction Set Computer) Machine) processor, the STMicroelectronics’ STM32L152, a battery management module, a high performance and high precision MEMS accelerometer sensing device, a built-in 12-bit analog-to-digital (AD) module, and a RTC real-time clock, which ensures low power consumption and high processing performance. The communication module is a CC1101 radio frequency (RF) chip, with 470M band and GFSK transmission mode. The gateway can be manipulated to send electromagnetic waves through the RF chip to activate the sensor for data collecting and communication when the sensor is dormant. The sampling frequency of the sensor is 50 Hz which is in accordance with the Specification for Bridge Structural Monitoring System (DG/TJ 08-2194-2016) [21] and can improve the data transmission quality and minimize the power consumption by using low-power awaken technology. The power supply of the sensor system adopts the LTC3331 power management chip. Considering the accuracy of the data acquisition of bridge acceleration, an ADXL345 sensing device is used in the system, which can make the system work with ultra-low electrical current consumption, 23–140 μA. The electrical current consumption of the sleep time is as low as 0.1 μA. The low power consumption mode supports the intelligent power management based on movement, thus it operates at a very low power consumption for threshold sensing and vibration acceleration measurement. The finished product of wireless acceleration sensor is shown in Figure 1 and Figure 2. 

Since the power supply for sensors is always a critical issue, a charge interface was designed in the wireless sensor, so the sensor can be recharged in case of an electricity shortage. In this study, the sensor uses the Fast Fourier Transformation (FFT) to convert the collected signals from time domain into frequency domain. Then the values of the signal in the frequency domain are arranged in descending order, and the first five points of the largest amplitude are extracted for sending to the gateway. The gateway then uploads the received data to the cloud platform. So the cloud platform can display the data composed of the five points in the frequency domain and the plots in the time domain through inverse FFT transformation, so the raw data is compressed by FFT. If needed, the original data collected by the wireless sensor can also be transmitted to the cloud platform through the gateway and uploaded to the database of the cloud platform.

#### 2.1.2. Performance Test

**Figure 3:**
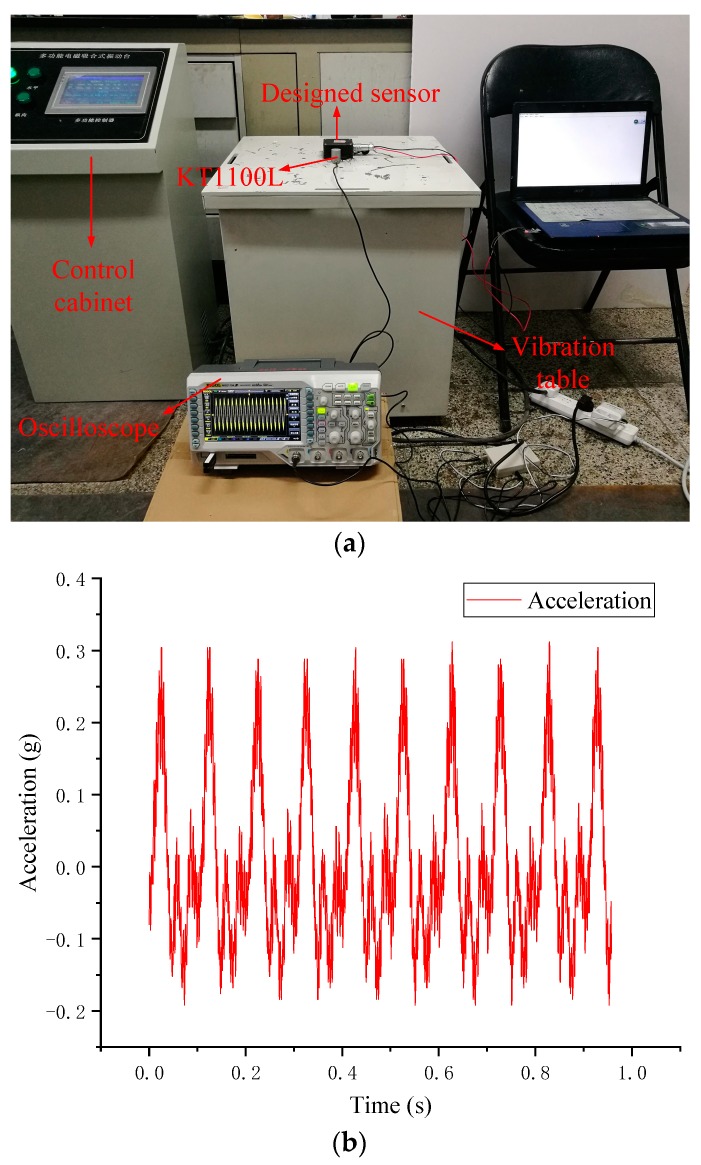

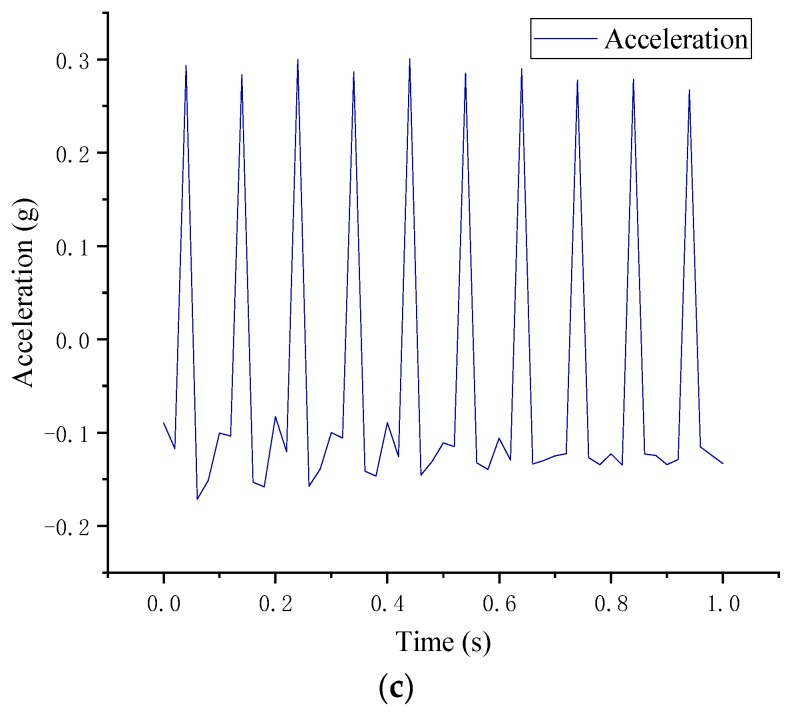
The acquired viberation: (**a**) the sensors tested at vibration table; (**b**) the data collected by KT1100L; (**c**) the data collected by designed wireless acceleration sensor.

In order to test the performance of the wireless acceleration sensor, a commercially available acceleration sensor manufactured by Yangzhou Ketu Co., Ltd. in Yangzhou City, Jiangsu Province, China, model KX-1100L, was purchased for the comparison study, which is a piezoelectric accelerometer with a sensitivity of 1000 mv/g, a frequency range of 0.5–5000 Hz, and the sampling frequency is 1000 Hz. The designed acceleration sensor and the KT-1100L were fixed together on a six-degree vibration table for testing, which was mainly composed of the table body and the control cabinet. During the test, the KT1100L acceleration sensor signal collector was a Tektronix DPO2024 oscilloscope where the number of bits was 12. The designed acceleration sensor transferred the data to the computer through a serial port. In the evaluation test, the control cabinet parameters (frequency and velocity) were adjusted to stimulate the vibration table, and the collected data of the two acceleration sensors within one second were extracted for comparison, which are depicted in Figure 3.

**Figure 4:**
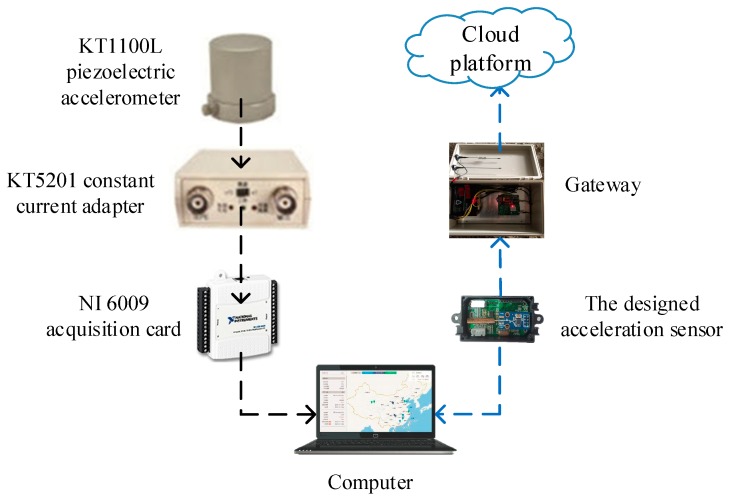
Comparison of data transmission modes of KT1100L and the designed wireless acceleration sensor.

As can be seen in Figure 3, the data of the designed acceleration sensor is basically consistent with those of the commercial sensor, but the designed acceleration sensor in this study had low energy consumption and cost, high integration, and quick data collection and transmission. The comparison between the designed sensor and the commercial sensor is shown in Figure 4.

The acceleration sensor designed in this study not only transmits the collected data directly to the computer through the data serial port, but also wirelessly transmits it to the gateway, and the gateway wirelessly transmits the data to the cloud platform. The KT1100L piezoelectric acceleration sensor need to be connected with the KT5201 constant current adapter and the NI6009 acquisition card to transmit data to the computer. The KT5210 constant current adapter has a frequency range of 0.3–10 KHz, an excitation voltage of 24 V and an excitation current of 2 mA. The NI6009 acquisition card has a resolution ratio of 14 bits, a sampling rate of 48 KHz. The acceleration sensor designed in this study is more convenient for data acquisition.

### 2.2. Wireless Cantilever Beam Vibration Sensor

**Figure 5:**
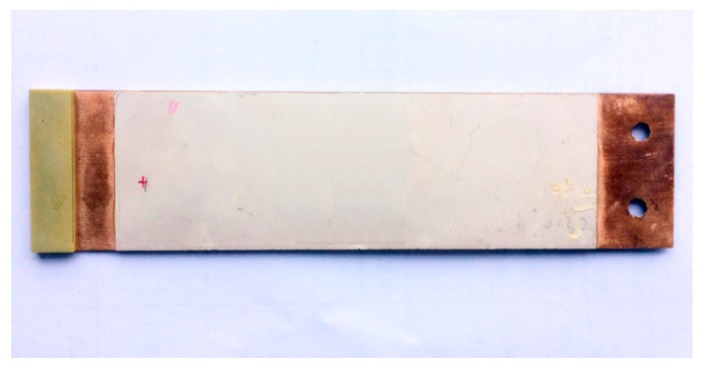
The manufactured cantilever beam.

In this paper, a piezoelectric cantilever beam, formally designed for energy harvesting, was optimized using the piezoelectric cantilever beam theory. The target natural frequency range of the sensor was selected to be 5–15 Hz based on the natural frequency and the loading frequency of most small and medium-sized bridges [22,23]. The optimal parameters for the cantilever beam was determined by simulating the effect of cantilever beam parameters on the natural frequency through the finite element method. The final beam dimensions are established to be 150 mm in length, 40 mm in width, 0.85 mm in total thickness, 0.35 mm thickness of the metal substrate, 0.25 mm thickness of the piezoelectric patches, and a 12 g mass block. The fabricated beam is shown in Figure 5.

A piezoelectric cantilever beam can generate an electric signal along with bridge vibration, so the electrical signal has a certain connection with the vibration of the bridge. In this paper, by connecting the cantilever beam with a data acquisition and transmission card innovatively, a wireless cantilever beam vibration sensor is developed, which has been successfully applied in bridge health monitoring. The cantilever beam of a sensor installed in a bridge will vibrate when a vehicle passes. There is a piezoelectric ceramic layer bonded on each side of the cantilever beam, which will generate analog signals with the vibration of the cantilever beam. The acquisition card then converts the analog signals into digital signals, and the wireless transmitter transmits the digital data with 470 M frequency band to a gateway. In the IoT system, the cantilever beam sensor can be used as a monitoring node, and the bridge vibration data can be transmitted wirelessly to the cloud platform through the gateway. So the data of bridge vibration can be analyzed in real-time with the collected data from the cloud platform [24].

**Figure 6:**
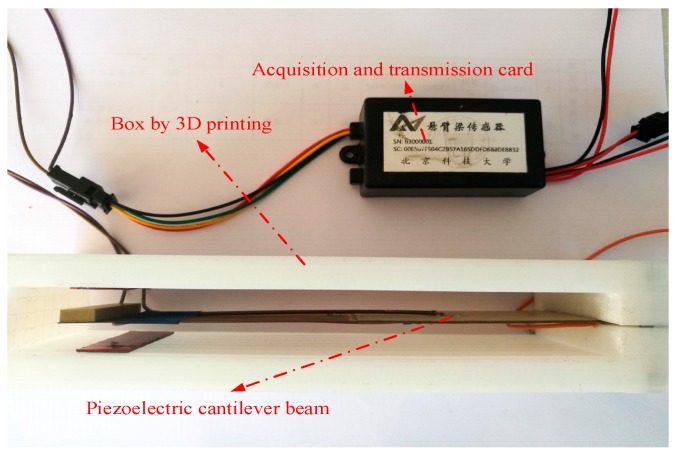
The fabricated wireless cantilever beam vibration sensor.

The packaging of the cantilever beam can facilitate installation of the sensor for bridge health monitoring, which is realized by 3D printing in this paper. The picture of a real product of the wireless cantilever beam vibration sensor is shown in Figure 6, where the welded wire on the upper and lower surface of the cantilever beam was connected with the acquisition and transmission card. The feasibility of data acquisition and wireless transmission of the wireless cantilever sensor designed in this paper will be verified in the application of the Internet of things system in field evaluations.

## 3. The Gateway

### 3.1. Design of the Gateway

**Figure 7:**
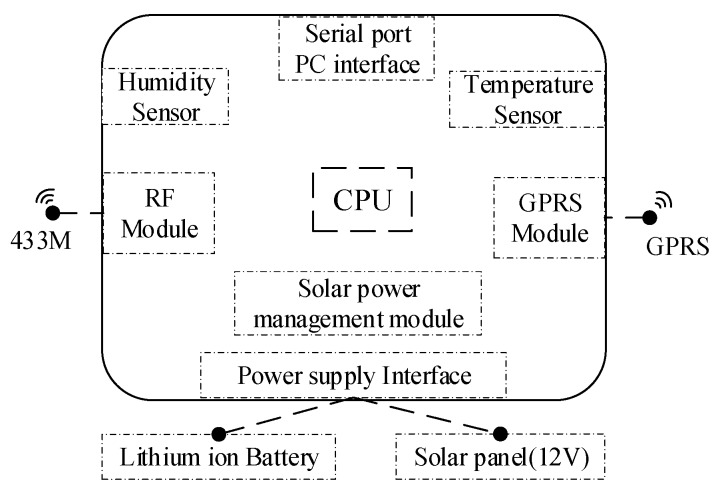
The gateway structure.

The gateway is the link between the server and the sensor, which is very important for remote monitoring. The communication of the gateway with the server applies the GPRS DTU module which contains a high-performance industrial-grade communication processor and industrial wireless modules. The data transmission between the gateway and the sensor is achieved by CC1101 in TI. The main controller is a STM32F103RCT6 processor with a high-performance ARM^®^ architecture™-M3 32-bit RISC core, and a built-in high-speed packet SRAM, which works at a voltage range of 2.0–3.6 V. The gateway can be powered by both external power and solar energy. The gateway also has multiple standards and an advanced communication interface, which can be easily connected to the temperature and humidity sensors for surrounding environment monitoring. The schematic diagram of the gateway structure is shown in Figure 7.

**Figure 8:**
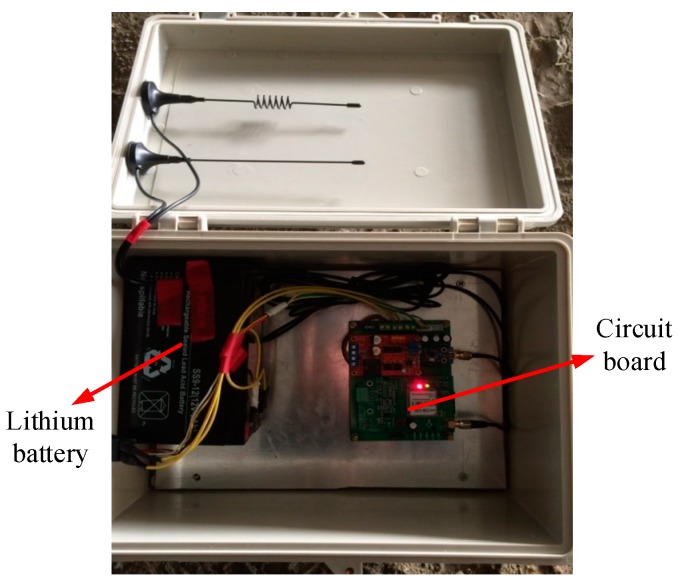
The photo of the gateway including lithium batteries and circuit boards.

In bridge transverse monitoring, one gateway is needed, while in longitudinal monitoring, the number of gateways depends on the bridge length. All sensors and gateways are numbered and the reference numbers of sensors and gateways are input into the cloud platform. When adding a gateway, the corresponding reference numbers of the sensors should be added to it. When the sensors are arranged on a bridge, the distance between the gateway and its corresponding sensors should be within 50 m. The gateway can use not only the built-in 12 V power, but also the outer power from solar panels. The solar panel management module for the gateway power supply has the solar energy maximum power point tracking (MPPT) function, which can realize automatic and intelligent charging management. The gateway is shown in Figure 8.

### 3.2. Communication Test

The gateway and the cloud platform communicate with the 4G stream, so the distance is not a problem. However, the gateway and the sensor communicate with the 470 M frequency band, which has a limited distance. Therefore, this study only tests the communication distance between the gateway and the sensor. In the test, the main index is the data packet loss rate (PLR) and the average time (T) the server received a data packet. *PLR* and T can be calculated by the following Equations (1) and (2).

(1) $$PLR=\frac{\left(N_{t}-N_{r}\right)}{N_{t}}\times 100\%$$

(2) $$T=\frac{\left(T_{N}{-T}_{1}\right)}{N-1}$$

where, $N_{t}$ is the number of packets sent by the sensing node; N is the number of packets received by the server; $T_{N}$ is the time when the server received the Nth packet; $T_{1}$ is the time when the server received the first packet.

**Figure 9:**
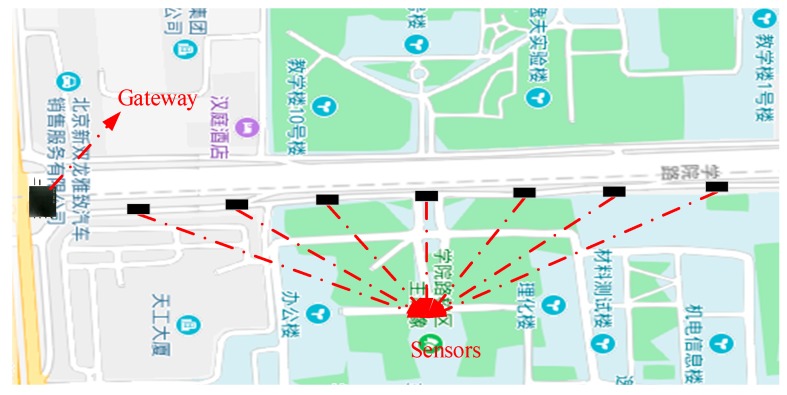
Layout of the gateway and the sensors.

The location of the sensor and gateway communication capability test is on the Xueyuan road, outside the west gate of the University of Science and Technology Beijing, Beijing, China. In this study, the gateway was placed at the northeast corner of the Xueyuan bridge, and a wireless sensor is placed each 100 m along the roadside. A total of 7 nodes were placed, as shown in Figure 9.

**Figure 10:**
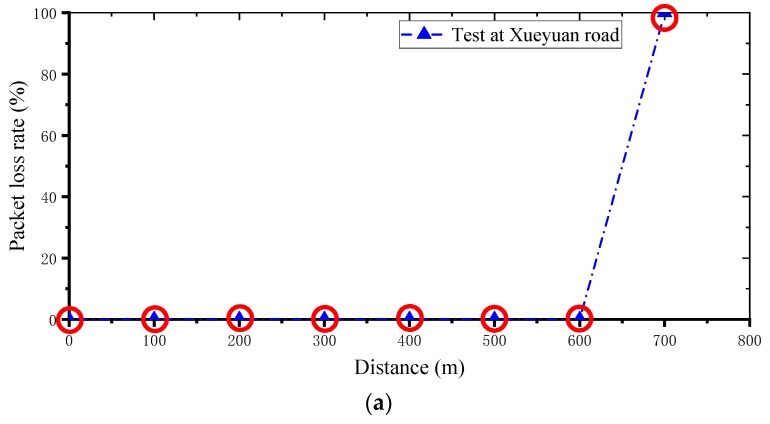

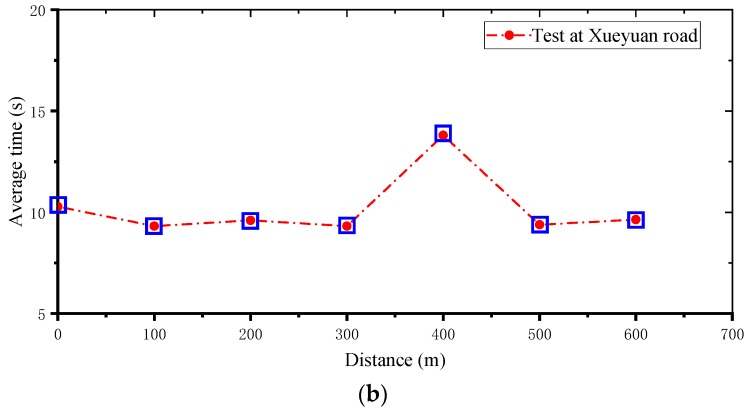
Validation results: (**a**) data packet loss rate; (**b**) average time the server received data packets.

The sensing node sends a certain number of data packets to the gateway at a fixed rate (0.1 Hz, or one data set at every 10 s) and at a fixed transmission power (20 dB). The number of test packets is 50, the results are shown in Figure 10, where the abscissa is the distance between the sensor and the gateway.

It can be seen from Figure 10a that the gateway can receive data from the sensors within a radius of 600 m. If it is more than 600 m, the data is completely lost. It can be seen from Figure 10b that the sensor node has a time delay at a distance of 400 m from the gateway, and the average time of other test points are basically the same. This may be because the node at 400 m is affected by nearby buildings or other communication equipment at that time. In future applications, gain modules can be added to the gateway to expand the communication range. In addition, in the sensor node layout of bridge monitoring, it is necessary to avoid interference of other communication devices and shield of other objects.

## 4. Design of Cloud Platform

**Figure 11:**
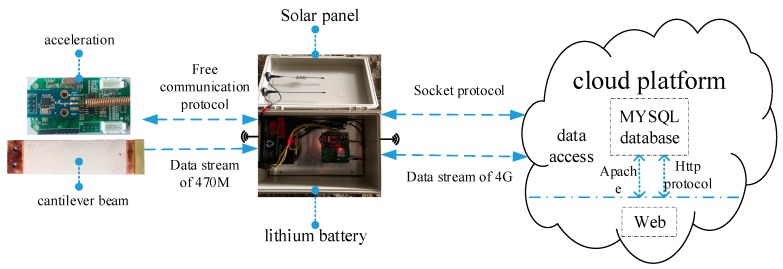
The technical service framework of the cloud platform.

The cloud platform, which is the terminal of the IoT system, is used to receive, store, analyze, and visualize data and manage the system front end. The cloud platform developed in this study uses the Alibaba cloud service as the background server with an Ubuntu12.04 system and a core of Linux system. The Apache HTTP Server is used as the web server with its efficiency, reliability and expansibility, which can compile the Perl/Python interpreter into the server convenient via an API extension. The MySQL is used as RDBMS (Relational Database Management System) to apply in WEB (World Wide Web). The Python language, which is one of the symbolic languages in the Linux system and can provide a rich module support for developing distributed applications conveniently, is used for sockets protocol development. The technical service framework of the cloud platform designed in this study is shown in Figure 11.

The Axure RP 7.0 is used to design the framework of the WEB end function prototype, which can rapidly create the framework, prototype and the specification document of the Web interface. The Sencha Architect 3 is used to develop the WEB end based on the design of the WEB end prototype, which includes database correlation, code edit of web page and web interaction. It is also convenient to design the required module function for bridge health monitoring including administrator login, data visualization, addition of sensor and gateway, monitoring status display, data storage and export.

**Figure 12:**
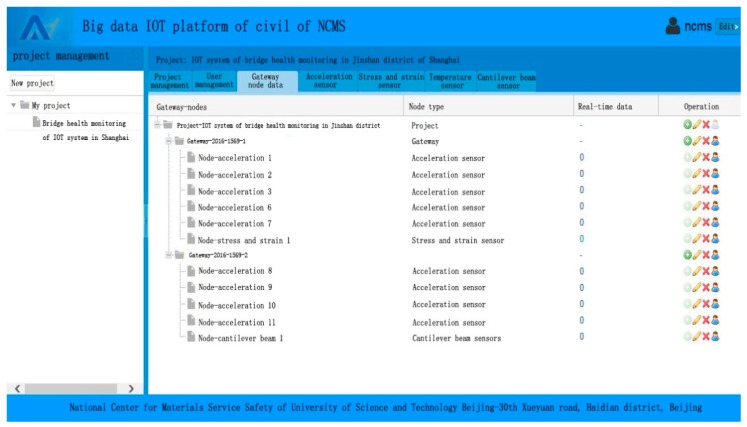
Interface of the cloud platform.

The cloud platform server and gateway use the Socket communication mechanism to insure the communication between different computers, and use the 4G communication technology to transfer data that can ensure the efficiency and high quality in bridge real-time monitoring. The gateway uses the protocol of the 470M frequency band to communicate with sensors, which can reduce the power consumption of the CPU. The main functions of the cloud platform include (1) user registration and login (administrator, client and designer); (2) project addition and management; (3) installation and management of gateways and sensors; (4) graphic display; (5) sub-interface function; (6) data check, storage and downloading; (7) real-time data analysis; (8) health assessment for monitored facilities; (9) early warning function of threshold value for monitored facilities; (10) other additional functions. The login interface of the cloud platform is shown in Figure 12.

## 5. Application of the IoT System in Bridge Health Monitoring

**Figure 13:**
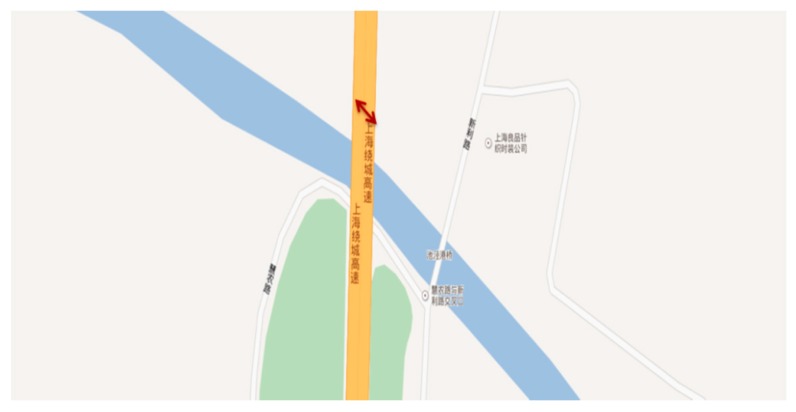
The location of the Chijing Bridge.

An integrated IoT system of bridge health monitoring is developed in this study through designing and fabricating the wireless sensor and the gateway, and the cloud platform according to the designs as described. A total of nine wireless acceleration sensors, a cantilever beam sensor and two gateways were assembled for field evaluations. These sensors and gateways are deployed on the cloud platform, and five acceleration sensors are deployed into the first gateway, four acceleration sensors and one cantilever beam sensors are deployed into the second gateway. The system and equipment were used for a demonstration test at Chijing Bridge of the G1501 Ring Expressway of Shanghai Jinshan district. Chijing Bridge is located in the southwest direction of the G1501 Ring Expressway, it is an assembly slab bridge, and has the left and right bridges. Each single bridge has 3 span continuous beams, and the spans are 20 m, 22 m and 20 m respectively. The width of the bridge is 21 m. There are 21 pieces of hollow slabs in the transverse direction. The location of the Chijing Bridge is shown in Figure 13, where the yellow line is the Chijing bridge and the double arrows is the position of the demonstration test. 

**Figure 14:**
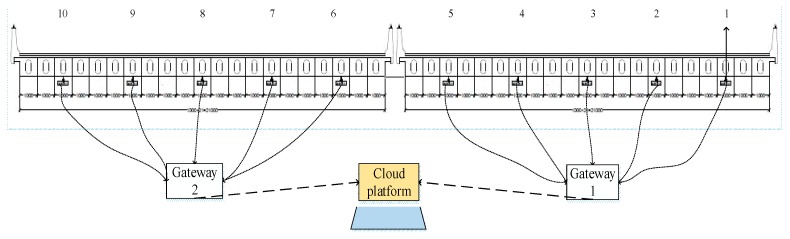
The layout sketch of the sensors and the gateway.

For bridges in service, one of the main factors affecting their health is weakening or failing of the transverse connections between beams, which leads to the decrease of the transverse collaborative work capability, and results in extremely unfavorable stress states in the upper structure of the bridge. Therefore, it is important to monitor the degradation of the lateral collaborative work capability. It might be possible to use acceleration data to evaluate bridge structural degradation through the deployment of smart sensors in an IoT network. Such monitoring exercises can also verify the effectiveness and practicability of the IoT platform designed for the bridge. The field trial monitoring was performed on one span of the Chijing Bridge. The vertical vibration acceleration of 10 plate beams was monitored at the transverse cross section of the middle span. The layout of sensors and the gateway is shown in Figure 14.

**Figure 15:**
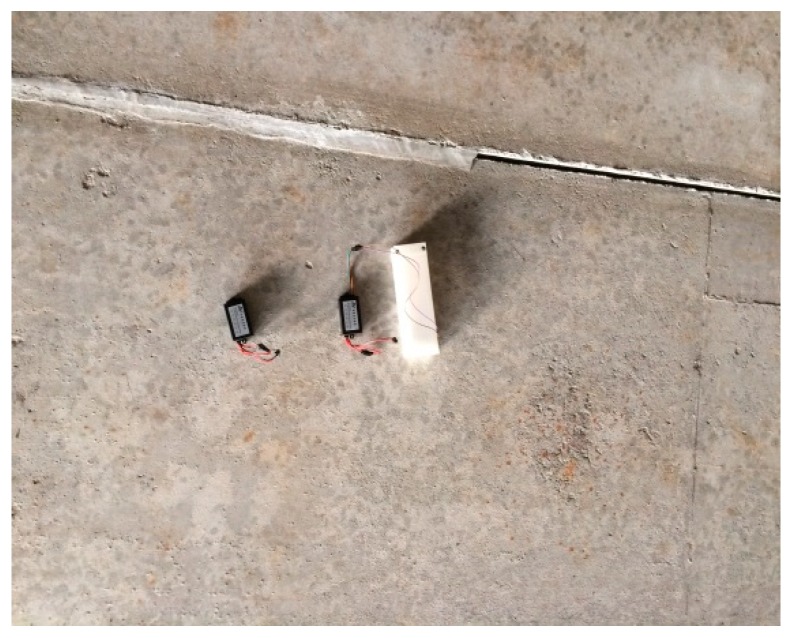
The sensor installation.

**Figure 16:**
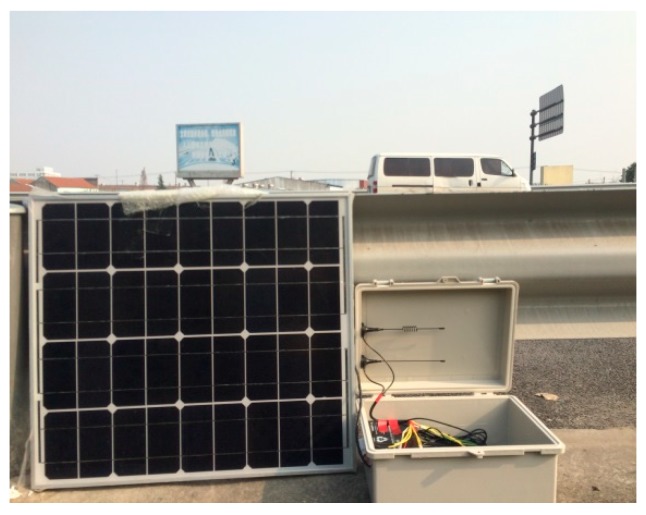
The gateway installation.

The wireless acceleration sensor and the wireless cantilever beam sensor designed in this study were installed underneath the deck of the Chijing Bridge. The gateway and the solar panels were deployed on the edge of the bridge. Vibration signal acquisition depends on the sensor position, therefore sensors were deployed to every three beam slabs in accordance with the characteristics of the Chijing bridge. A total of ten sensors have been deployed. So the health conditions of the bridge can be analyzed based on the collected data. The sensor and gateway installations are shown in Figure 15 and Figure 16.

**Figure 17:**
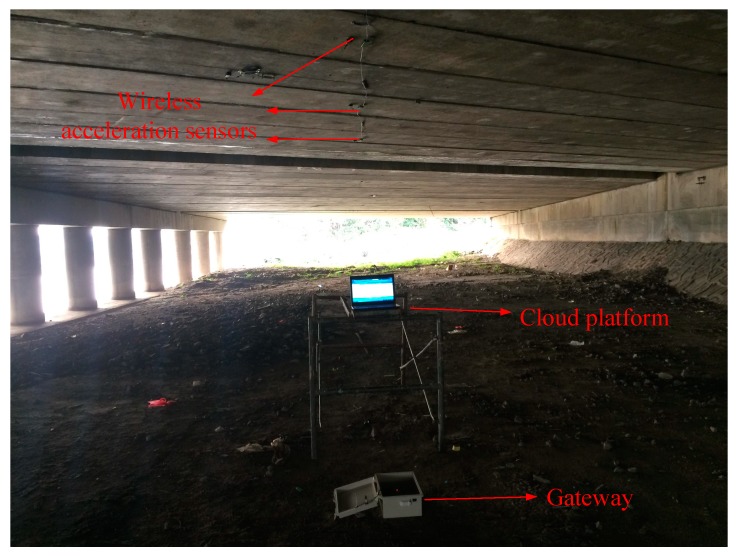
The onsite layout of the IoT system for bridge health monitoring.

During monitoring, the sensors collect data and communicate with the gateway automatically. Managers can use a computer to login to the cloud platform to view the data in any place. In this study, we accessed the cloud platform for real-time data monitoring on the bridge site in order to facilitate on-site adjustment. The site layout of the IoT system for the bridge health monitoring is shown in Figure 17. As can be seen in this figure, the bottom of the bridge beam is instrumented with sensors. The gateway (the white box on the ground), and the computer (in the middle) that is visiting the cloud platform for monitoring management are also shown in Figure 17. In this system, the sensors wirelessly transmit data to the gateway, and the gateway wirelessly transmits the data to the cloud platform. The users can view the data by logging into the cloud platform from a computer.

**Figure 18:**
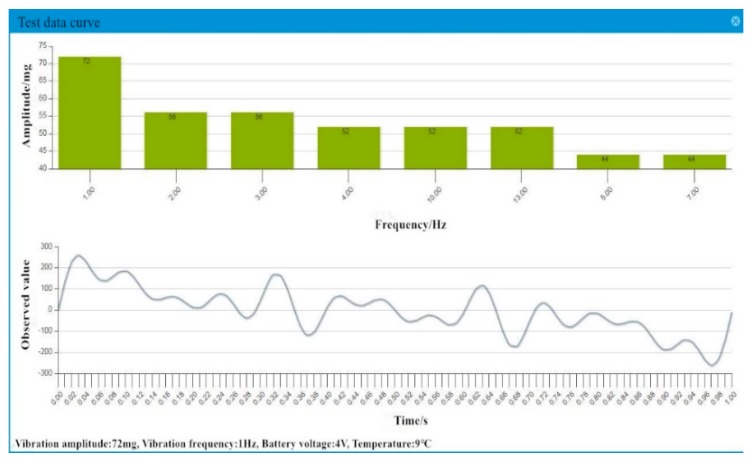
The time domain and frequency domain of monitoring.

Through the IoT system of the Chijing Bridge, all the raw data collected in real-time by the sensor were saved in the cloud platform database. Then the data were processed and analyzed to judge whether the indicators are beyond reasonable range. On the cloud platform, all the raw data can be accessed, so are the data in time domain and in frequency domain. Figure 18 depicts the monitored vibration data of a second in frequency domain and in time domain. 

**Figure 19:**
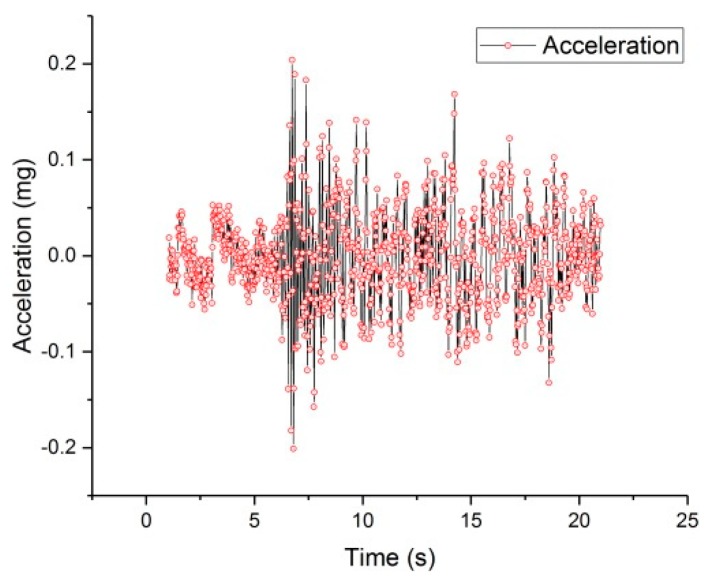
The data of twenty seconds.

**Figure 20:**
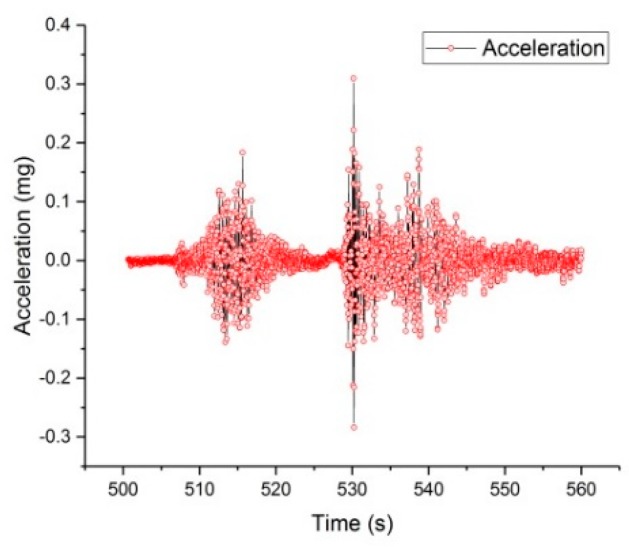
The data of one minute.

**Figure 21:**
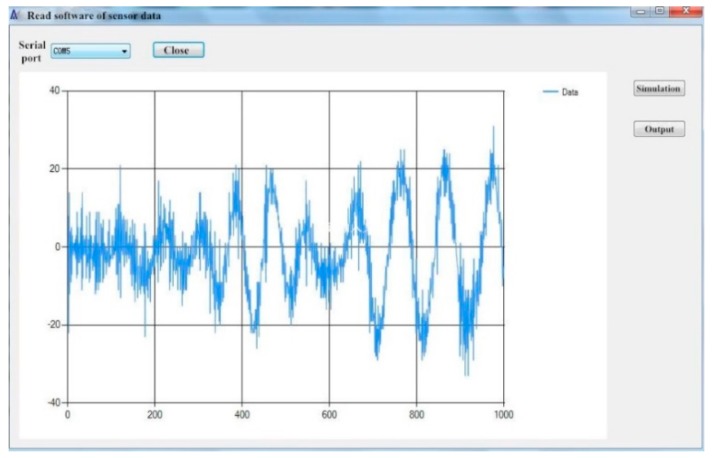
Data of cantilever sensor.

A typical period of monitored data was extracted from the continuous monitored data collected by the IoT system to illustrate the vibrations of the bridge subjected to the traffic loading, which are depicted in Figure 19 and Figure 20. Figure 21 depicts the typical data collected by the wireless cantilever sensor. As shown in the figures, the bridge vibrations are consistent with the traffic loadings. The data demonstrated that the entire system can work coherently. 

The influences of vehicle dynamic loading on the bridge vibration are intuitively shown in the figures. The speed and displacement of the bridge beam can be calculated according to the collected data. It should be noted that the wireless cantilever beam sensor has less noises than the commercial sensors, which also verifies the success of the wireless cantilever sensor in data acquisition and wireless transmission. The successful application of the piezoelectric ceramic based cantilever sensor lays a good foundation for the development of self-powered sensors. This paper tests the practicability of wireless acceleration sensors, wireless gateway and cloud platform through the IoT system of the Chijing Bridge. The application of the IoT based real-time health monitoring system offers a potential for the safety of the bridge structure, the vehicles, and the pedestrians. And the system is helpful for the bridge to achieve longer service life.

## 6. Summary

In this paper, an IoT system for bridge vibration monitoring was developed via integration of the wireless sensors, the gateways, and the cloud platform, which can realize the remote cluster monitoring of the bridge in real-time. The validity of the IoT based system in bridge health monitoring was demonstrated at the Chijing Bridge in Shanghai. The discoveries of the paper are summarized as follows:

1. The wireless sensor based on microelectronics technology has smaller dimensions for convenient installations, which can reduce the costs of installation and maintenance. It can be recharged with solar panels or a piezoelectric cantilever beam to ensure long-term monitoring.
2. The gateway can accommodate multiple data collections for various built-in sensors such as the temperature and humidity sensors which can be used for real-time monitoring of the surrounding environmental conditions. All the monitored data can be transmitted and saved in the database of the cloud platform, which is convenient for remote access.
3. The IoT system can avoid the disadvantages of wiring and power supply of traditional monitoring, and also avoid interruption of regular bridge inspection. Meanwhile the installation of the sensor and gateway is convenient, which can save manpower and resources. The system can also meet the requirements of bridge clusters monitoring due to its convenient wireless communication framework.

In future research, the service performance of the wireless acceleration sensor will be evaluated to verify its service life and probability of malfunction. The data communication between the wireless sensor and gateway will be strengthened by adding a gain module. The structure of the piezoelectric cantilever beam will be further optimized to improve the power output so that a self-powered sensor could be formed by combining the wireless cantilever sensor describing in Section 2.2.

The IoT system was mainly applied in the new bridge, and the monitoring time is relatively short. Experiments on bridge damage monitoring will be conducted in the near future.
